# An interactive key to the *Chrysochromulina* species (Haptophyta) described in the literature

**DOI:** 10.3897/phytokeys.34.6242

**Published:** 2014-02-13

**Authors:** Marie-Josèphe Chrétiennot-Dinet, Nicolas Desreumaux, Régine Vignes-Lebbe

**Affiliations:** 1 Sorbonne Universités, UPMC Univ Paris 06, UMS 2348, Observatoire Océanologique, F-66650 Banyuls/Mer, France; 2 Sorbonne Universités, UPMC Univ Paris 06, UMR 7207 (MNHN, UPMC, CNRS) et UMR 7205 (MNHN, UPMC, CNRS, EPHE), CP48, 57 rue Cuvier, 75231 Paris cedex 05, France

**Keywords:** Interactive key, identification tool, XPER², web application, morphology, description protocol, phytoplankton, *Chrysochromulina*

## Abstract

We present a general overview of features and technical specifications of an original interactive key web application for the identification of *Chrysochromulina* species. The list of species, originally described as belonging in the genus *Chrysochromulina*, is given and recent taxonomic changes in species and genera of the order Prymnesiales are provided. We briefly discuss the interest of such a key for the identification of phytoplanktonic species.

## Introduction

The genus *Chrysochromulina*, erected by Lackey (1939) is an important component of the marine and brackish phytoplankton although the type species occured in fresh water. Electron microscopy (EM) has been a key tool for a specific identification and Mary Parke & Irene Manton were pioneers in reviewing the type species (Parke et al. 1962) and describing more than ten species between 1955 and 1966. They remained for many years the specialists of the genus until Barry Leadbeater added some more new species so that almost half the number of species known today were described by 1974. With the extent of TEM (Transmission Electron Microscopy) or SEM (Scanning Electron Microscopy) studies, the genus appeared worldwide distributed and some species were found to produce massive blooms, some of which were eventually toxic (Moestrup 1994). The 1988 bloom of *Chrysochromulina polylepis* Manton & Parke (Dahl et al. 1989) was the first event of toxic bloom causing important economic impact, raising a considerable interest of the scientific community especially in Scandinavia. Two PhD thesis were submitted (Jensen 1998, Eikrem 1999 ) with an illustrated key for identification of species of this genus, based on morphological characters (Eikrem et al. 1998). With the 21th century molecular biology introduced changes in the delineation of classes and orders and the genus *Chrysochromulina* was considered as polyphyletic (Edvardsen et al. 2000).

The class name Haptophyceae was first used by Christensen in 1962 but Hibberd introduced the typified class name Prymnesiophyceae (Hibberd 1976), both names being considered as valid. More recently Silva et al. (2007) advise the use of the name Coccolithophyceae for this class, considering that the class name Coccolithophyceae Rothmaler 1951 had priority over Haptophyceae and Prymnesiophyceae. However this class name remains a matter of debate and therefore is not mentionned in the title.

Within the class, the genus *Chrysochromulina* was for a long time placed in the order Prymnesiales and the family Prymnesiaceae. However, from DNA phylogenies and morphological comparisons, Edvardsen et al. (2011) reviewed the taxonomy of the Prymnesiales. They emended the Family Prymnesiaceae W. Conrad ex O.C. Schmidt emend. Edvardsen, Eikrem & Medlin, (Edvardsen et al. 2011), placing five species of *Chrysochromulina* (*Chrysochromulina palpebralis*, *Chrysochromulina polylepis*, *Chrysochromulina kappa*, *Chrysochromulina chiton* and *Chrysochromulina minor*) in the genus *Prymnesium*, and five other species (*Chrysochromulina brevifila*, *Chrysochromulina ericina*, *Chrysochromulina fragaria*, *Chrysochromulina herdlensis* & *Chrysochromulina hirta*) in the new genus *Haptolina* Edvardsen & Eikrem (Edvardsen et al. 2011). An unnamed species, cited as *Chrysochromulina* sp4 (Eikrem & Edvardsen, 1999), is considered as the type species of the new genus *Pseudohaptolina* Edvardsen & Eikrem. They give a formal description of this species as *Pseudohaptolina arctica* Edvardsen & Eikrem (Edvardsen et al. 2011). Because of these changes, the family Chrysochromulinaceae Edvardsen, Eikrem & Medlin is now restricted to the unique genus *Chrysochromulina* with the remaining species, all being saddle-shaped cells (Edvardsen et al. 2011).

As we are dealing here with an identification key, we have taken into consideration all species originally described as *Chrysochromulina* in the literature (or moved to this genus as for *Chrysocampanula spinifera* (Fournier) by Pienaar and Norris in 1979) but modifications of their taxonomic status are mentioned in the species descriptions. References are restricted to papers giving the original description of a species or an emended description.

## Project description

### Taxonomic coverage

The key covers 58 species originally described as *Chrysochromulina*. References for publications dealing with their description and occurrence are given. A detailed description is provided and illustrations of a whole cell as well as for the different scale types, in some cases from unpublished material seen in SEM, are included. It is noticeable that two of them have different morphologies described as “forma”: *Chrysochromulina polylepis* , now *Prymnesium polylepis*, “authentic” or “alternate” (Edvardsen and Paasche 1992, Edvardsen et al. 1996, Edvardsen and Medlin 1998); *Chrysochromulina palpebralis* f. *palpebralis* or *Chrysochromulina palpebralis* f. *bisquamata* (Seoane et al. 2009). As mentioned before, an additional species, referred to as *Chrysochromulina* sp. 4 (Eikrem and Edvardsen 1999) is now considered as the type species of the new genus *Pseudohaptolina* (Edvardsen et al. 2011). The terminal taxa of the key are 63 because all morphological forms are treated separately. A few of them (freshwater species) are poorly described (*Chrysochromulina inornamenta* Wujek & Gardiner, *Chrysochromulina chiton* var. *minuta* and *Chrysochromulina papillata* Gao, Tseng & Guo, *Chrysochromulina laurentiana* Kling) but still may be identified through this key.

### List of the terminal taxa included in the current version of the database (last update, July 2013)

*Chrysochromulina acantha* Leadbeater & Manton (1971); *Chrysochromulina adriatica* Leadbeater (1974); *Chrysochromulina arenghotii* Jensen & Moestrup (1999); *Chrysochromulina alifera* Parke & Manton (1956) in Parke et al. (1956); *Chrysochromulina apheles* Moestrup & Thomsen (1986); *Chrysochromulina bergenensis* Leadbeater (1972); *Chrysochromulina birgeri* Hällfors & Niemi (1974); *Chrysochromulina brachycylindra* Hällfors & Thomsen (1985); *Chrysochromulina brevifilum* Parke & Manton (1955) in Parke et al. (1955), now *Haptolina brevifila* (Parke & Manton) Edvardsen & Eikrem 2011; *Chrysochromulina breviturrita* Nicholls (1978); *Chrysochromulina camella* Leadbeater & Manton (1969); *Chrysochromulina campanulifera* Manton & Leadbeater (1974); *Chrysochromulina chiton* Parke & Manton (1958) in Parke et al. (1958), now *Prymnesium chiton* (Parke & Manton) Edvardsen, Eikrem & Probert (2011); *Chrysochromulina chiton* var.* minuta* Gao, Tseng & Guo (1993); *Chrysochromulina cyathophora* Thomsen (1979); *Chrysochromulina cymbium* Leadbeater & Manton (1969); *Chrysochromulina discophora* Manton (1983); *Chrysochromulina elegans* Estep et al. (1984); *Chrysochromulina ephippium* Parke & Manton (1956) in Parke et al. (1956); *Chrysochromulina ericina* Parke & Manton (1956) in Parke et al. (1956), now *Haptolina ericina* (Parke & Manton) Edvardsen & Eikrem 2011; *Chrysochromulina fragaria* Eikrem & Edvardsen (1999), now *Haptolina fragaria* (Eikrem & Edvardsen) Edvardsen & Eikrem 2011; *Chrysochromulina fragilis* Leadbeater (1972); *Chrysochromulina herdlensis* Leadbeater (1972), now *Haptolina herdlensis* (B. Leadbeater) Edvardsen & Eikrem 2011; *Chrysochromulina hirta* Manton (1978b), now *Haptolina hirta* (Manton) Edvardsen & Eikrem 2011; *Chrysochromulina inornamenta* Wujek & Gardiner (1985); *Chrysochromulina kappa* Parke & Manton (1955) in Parke et al. (1955), now *Prymnesium kappa* (Parke & Manton) Edvardsen, Eikrem & Probert, 2011; *Chrysochromulina lanceolata* Chrétiennot-Dinet, Nezan & Puigserver (2003) in Puigserver et al. (2003); *Chrysochromulina latilepis* Manton (1982); *Chrysochromulina laurentiana* Kling H.J. (1981); *Chrysochromulina leadbeateri* Eikrem & Throndsen (1998); *Chrysochromulina limonia* Jensen & Moestrup (1998); *Chrysochromulina mactra* Manton (1972); *Chrysochromulina mantoniae* Leadbeater (1972) ; *Chrysochromulina megacylindra* Leadbeater (1972); *Chrysochromulina microcylindra* Leadbeater (1972); *Chrysochromulina minor* Parke & Manton (1955) in Parke et al. (1955), now *Prymnesium minus* (Parke & Manton) Edvardsen, Eikrem & Probert, 2011; *Chrysochromulina novae-zelandiae* Moestrup (1979); *Chrysochromulina orbiculata* Rouchijajnen (1972); *Chrysochromulina pachycylindra* Manton & Oates (1981) in Manton et al. (1981); *Chrysochromulina palpebralis* Seoane, Eikrem, Edvardsen & Pienaar (2009), now *Prymnesium palpebrale* (Seoane, Eikrem, Edvardsen & Pienaar) Edvardsen, Eikrem & Probert, 2011; *Chrysochromulina papillata* Gao, Tseng & Guo (1993); *Chrysochromulina parkae* Green & Leadbeater, (1972); *Chrysochromulina parva* Lackey (1939); *Chrysochromulina pelagica* Estep, Davis, Hargraves & Sieburth (1984); *Chrysochromulina planisquama* Hu & Tseng (2005) in Hu et al. (2005); *Chrysochromulina polylepis* Manton & Parke (1962), now *Prymnesium polylepis* (Manton & Parke) Edvardsen, Eikrem & Probert 2011; *Chrysochromulina pontica* Rouchijajnen (1966); *Chrysochromulina pringsheimii* Parke & Manton (1962); *Chrysochromulina pseudolanceolata* Chrétiennot-Dinet & Puigserver (2003) in Puigserver et al. (2003); *Chrysochromulina pyramidosa* Thomsen (1977); *Chrysochromulina quadrikonta* Kawachi & Inouye (1993); *Chrysochromulina rotalis* Eikrem & Throndsen (1999); *Chrysochromulina scutellum* Eikrem & Moestrup (1998); *Chrysochromulina simplex* Estep, Davis, Hargraves & Sieburth (1984) emend. Birkhead and Pienaar (1995); *Chrysochromulina spinifera* (Fournier 1971) Pienaar & Norris (1979) back to *Chrysocampanula spinifera* Fournier 1971 (see Edvarsen, Eikrem & Probert, 2011); *Chrysochromulina strobilus* Parke and Manton (1959) in Parke et al. (1959); *Chrysochromulina tenuispina* Manton (1978a); *Chrysochromulina tenuisquama* Estep, Davis, Hargraves & Sieburt (1984); *Chrysochromulina throndsenii* Eikrem (1996); *Chrysochromulina vexillifera* Manton & Oates (1983).

### Characters used in the key

The key matrix is based on one ecological character (habitat) and 19 morphological descriptors seen in light or electron microscopy, under live conditions or after fixation for EM observations. They range from cell shape to scale ornamentation. Details of scales can be obtained by specific techniques with TEM, such as direct preparations (Moestrup and Thomsen 1980, Jensen 1998) and cultures are generally needed. Although rarely used, SEM can also provide interesting results with natural samples (Puigserver et al. 2003). For fragile cells, 3 mL of sample are fixed with 50 µL of a 1:1 Lugol/Glutaraldehyde (25%) solution, centrifuged on a Thermanox cell culture coverslip coated with poly-L-lysine (0.1%) for a better adherence of cells, critical point dried and then examined with a field emission scanning electron microscope.

### List of descriptors used in the key:

HABITAT: marine, brackish, freshwater

SHAPE: spherical-subspherical, elongate to round, lanceolate, saddle-shaped

CELL LENGTH, CELL WIDTH: min. and max. sizes are given for each form or species.

FLAGELLA: Two flagella are present and may be equal or sub-equal, in that case the length of the longer and shorter flagellum are given.

HAPTONEMA BEHAVIOR: coiling, rarely coiling, non coiling.

HAPTONEMA LENGTH: min. and max. size (in some cases, the haptonema may be very long)

NUMBER OF SCALE TYPES: in some cases, scales may be displayed in several layers and show up to four different types but there is always a layer of plate scales as cell covering.

SCALE APPENDICES: besides plate scales, a number of different appendices can be observed : spine, cylinder or another typical ornamentation.

PLATE SCALE LENGTH AND WIDTH: min. and max. sizes are given for all plate scales.

### Software used

The interactive key is developed using Xper2 version 2.2 software. It is free software available with multilingual interface and compatible with different OS (Windows, MacOS and Linux) under a creative commons license (BY-CC-ND). You can download it on http://www.infosyslab.fr and find on this website a complete documentation with technical details, user manual and knowledge bases.

Xper2 offers an editor to structure and analyse descriptive data and an interface for interactive free access key (Ung et al. 2010). Keys of various taxonomic groups are already available with Xper2 (Kerner et al. 2011) (Mathieu et al. 2012) (Thomas and De Franceschi 2013).

We installed the interactive key on a web server with Apache2, choosing the English interface. This content is under a creative commons license (BY-CC-ND), except when a special information is attached to images.

### The knowledge base

Xper2 manages structured descriptive data: all the terminal taxa of the key are described using the same terms (descriptor and character states labels), and so the taxa can be compared automatically.

**Figure 1.**
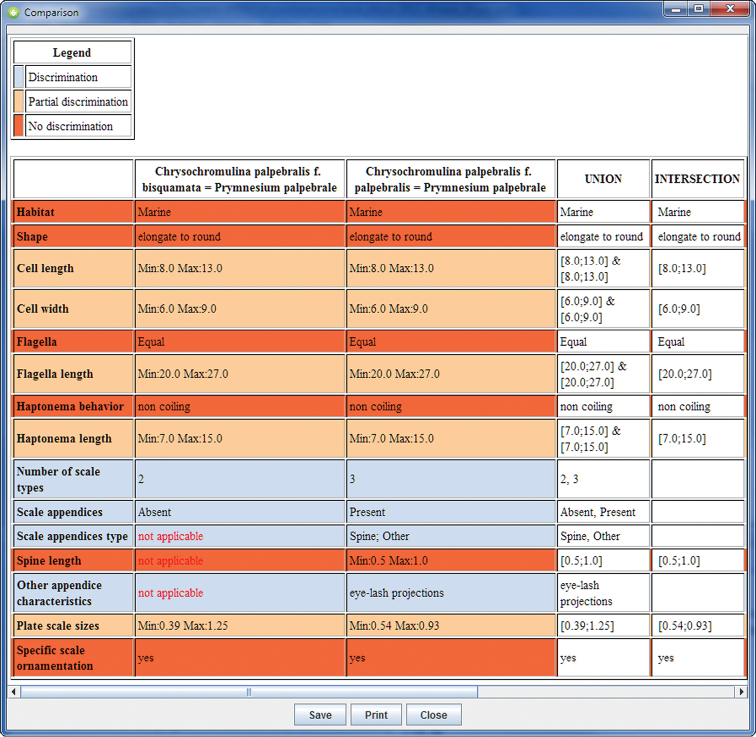
Comparison of the two forms of *Prymnesium palpebrale*. The two forms are very similar and differ only on scales and appendices.

Figure 1 presents the comparison of the two forms attributed to *Prymnesium palpebrale* (previously *Chrysochromulina palpebralis*). The different colors allow to point easily where the descriptions are distinct, overlap, or are the same. Here the two forms differ on scale type number and appendices.

**Figure 2.**
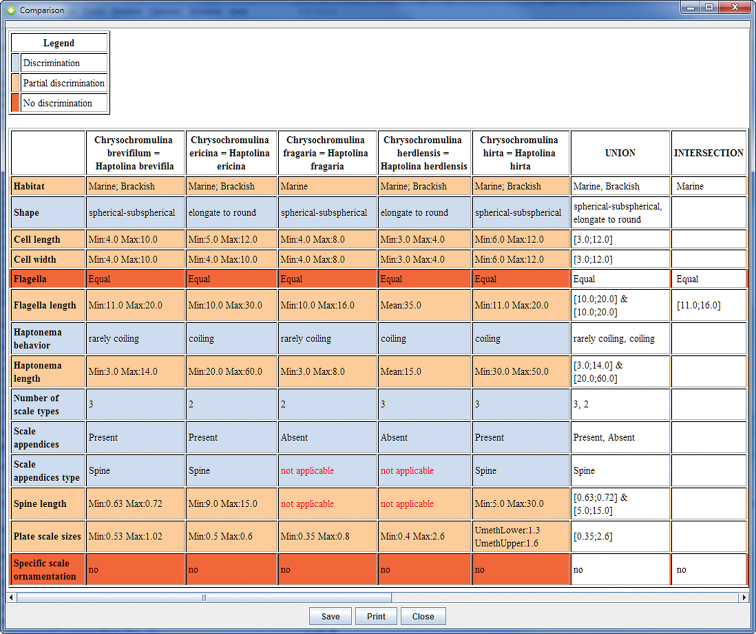
Comparison of the five species of the new genus *Haptolina*. The column «intersection» on the right side gives the values shared by the five species.

In the same way the comparison of the five species (*Haptolina brevifila*, *Haptolina ericina*, *Haptolina fragaria*, *Haptolina herdlensis* and *Haptolina hirta*) previously known as *Chrysochromulina* but attributed in 2011 in the new genus *Haptolina* (Edvardsen et al. 2011) shows that these species share few attributes used in the key (Figure 2).

### The online interactive key

Our key of the *Chrysochromulina* species is a free access key accessible at http://www.obs-banyuls.fr/chrysochromulina . It offers an interactive and flexible way to identify these phytoplanktonic species.

A classical polytomous key consists of a series of questions (characters), each one offering alternative statements (Hagedorn et al. 2010). A free access key is a more flexible identification key: the sequence of choices is defined by the user preventing these of characters difficult or impossible to observe.

**Figure 3.**
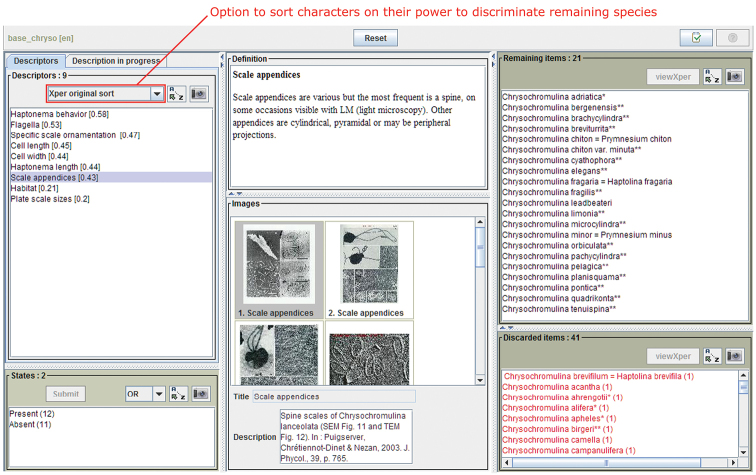
Example of screen of the free access key. On the left the user chooses descriptor and states with the help of the additional ressources, text and images in the center of the screen. On the right the lists of remaining and eliminated species.

Figure 3 shows the screen during an identification process. Each item (terminal taxon of the key) is documented by a text including nomenclatural data, type locality, literature references and morphological data, and is illustrated by several images. Descriptors and character states are also documented and illustrated.

**Figure 4.**
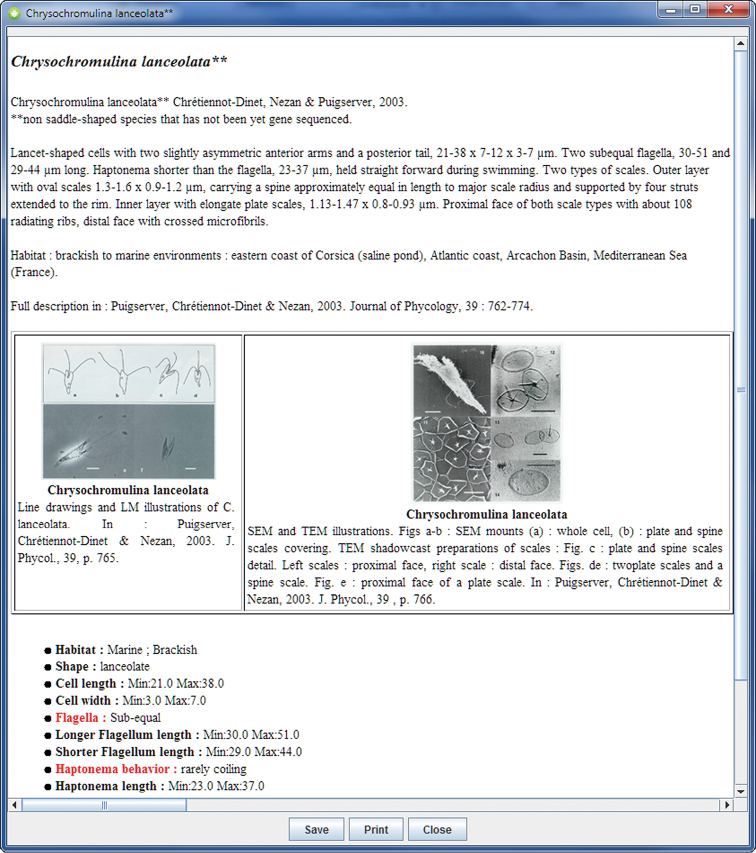
Example of descriptors (in red) that allow differentiation between *Chrysochromulina lanceolata* and a specimen under identification.

At each step, the user may ask the software to find the best characters to distinguish the possible taxa. Three different measures are proposed Xper, Jaccard, and Sokal & Michener (as ”Best descriptors” in the select box). For each pair of remaining taxa, each coefficient measurement compares the possible states and the final result is the sum for all the pairs. Xper coefficient checks only if there is no overlap (it means the two taxa may be completely distinct on this character) and so the measure for one pair of taxa is 0 (if overlapping) and 1 (if no overlapping). Jaccard coefficient was initially developed to compare sets of binary characters; here the states are considered as the binary characters and the comparison takes into account the ratio between the number of states possible for only one taxon of the pair and the number of states possible for at least one taxon of the pair. In the Sokal & Michener coefficient, the states which are not possible for the two taxa are also taken into account. The three measures are described in Burguière et al. (2013).

## Conclusions

Identification of *Chrysochromulina* species has long been reserved to specialists as it is a major difficulty for most phytoplanktonologists. The cells are very small, often overlooked or placed as “unidentified” species in field studies. Cultures and specific preparations are generally needed to get relevant information on morphological features. A key for identification of Scandinavian species (Eikrem et al. 1998), based on TEM observations of cultures has only been published in PhD Theses (Jensen1998 and Eikrem 1999). A list of species as part of toxic haptophytes was published by Moestrup & Thomsen in a manual on harmful marine microalgae (Moestrup and Thomsen 2004). However original descriptions are not always available for researchers. In this interactive key, all species found in the literature are treated and information necessary for their identification is provided. This key is a very powerful tool for a taxonomic work on the genus and is therefore strongly recommended, especially for phytoplanktonologists working on nanoflagellates. The content of the key was carefully checked and tested with information on species characteristics found in the Scandinavian key (Jensen 1998, Eikrem 1999) and unpublished data obtained by one author (M-J C-D). Among all descriptors used in this key, those concerning the scale description are most important and the mean number of required characters to identify a species is 4.3. SEM preparations from field samples seem promising for identification of these species (Puigserver et al. 2003 and unpublished illustrations shown in the key). The choice of characters introduced without order is also an important advantage as compared to a classical key: characteristics of a typical scale may be enough for a specific identification.
